# Genome Plasticity and Polymorphisms in Critical Genes Correlate with Increased Virulence of Dutch Outbreak-Related *Coxiella burnetii* Strains

**DOI:** 10.3389/fmicb.2017.01526

**Published:** 2017-08-10

**Authors:** Runa Kuley, Eric Kuijt, Mari A. Smits, Hendrik I. J. Roest, Hilde E. Smith, Alex Bossers

**Affiliations:** ^1^Department of Infection Biology, Wageningen Bioveterinary Research Lelystad, Netherlands; ^2^Host Microbe Interactomics, Wageningen University and Research Centre Wageningen, Netherlands; ^3^Department of Bacteriology and Epidemiology, Wageningen Bioveterinary Research Lelystad, Netherlands

**Keywords:** *C*. *burnetii*, Q fever, whole genome sequencing, comparative genomics, virulence, transposons, mutations, orthologs

## Abstract

*Coxiella burnetii* is an obligate intracellular bacterium and the etiological agent of Q fever. During 2007–2010 the largest Q fever outbreak ever reported occurred in The Netherlands. It is anticipated that strains from this outbreak demonstrated an increased zoonotic potential as more than 40,000 individuals were assumed to be infected. The acquisition of novel genetic factors by these *C. burnetii* outbreak strains, such as virulence-related genes, has frequently been proposed and discussed, but is not proved yet. In the present study, the whole genome sequence of several Dutch strains (CbNL01 and CbNL12 genotypes), a few additionally selected strains from different geographical locations and publicly available genome sequences were used for a comparative bioinformatics approach. The study focuses on the identification of specific genetic differences in the outbreak related CbNL01 strains compared to other *C. burnetii* strains. In this approach we investigated the phylogenetic relationship and genomic aspects of virulence and host-specificity. Phylogenetic clustering of whole genome sequences showed a genotype-specific clustering that correlated with the clustering observed using Multiple Locus Variable-number Tandem Repeat Analysis (MLVA). Ortholog analysis on predicted genes and single nucleotide polymorphism (SNP) analysis of complete genome sequences demonstrated the presence of genotype-specific gene contents and SNP variations in *C. burnetii* strains. It also demonstrated that the currently used MLVA genotyping methods are highly discriminatory for the investigated outbreak strains. In the fully reconstructed genome sequence of the Dutch outbreak NL3262 strain of the CbNL01 genotype, a relatively large number of transposon-linked genes were identified as compared to the other published complete genome sequences of *C. burnetii*. Additionally, large numbers of SNPs in its membrane proteins and predicted virulence-associated genes were identified in all Dutch outbreak strains compared to the NM reference strain and other strains of the CbNL12 genotype. The presence of large numbers of transposable elements and mutated genes, thereof most likely resulted in high level of genome rearrangements and genotype-specific pathogenicity of outbreak strains. Thus, the epidemic potential of Dutch outbreak strains could be linked to increased genome plasticity and mutations in critical genes involved in virulence and the evasion of the host immune system.

## Introduction

*Coxiella burnetii* is the pathogenic agent of Q fever which is a zoonotic infectious disease (Maurin and Raoult, 1999). It is an obligate intracellular gram-negative bacterium, which thrives within the acidic parasitophorous vacuole (PV) of eukaryotic cells (Akporiaye et al., 1983). Domestic ruminants such as goats, sheep and cattle are usually the primary reservoir for the *C. burnetii* strains causing Q fever in humans (Woldehiwet, 2004). The main clinical symptoms of Q fever widely differ between hosts (Maurin and Raoult, 1999; Norlander, 2000; Woldehiwet, 2004). Abortions are usually manifested in goats (Palmer et al., 1983; Roest et al., 2011a), whereas clinical symptoms are rarely observed in cattle (To et al., 1998; Arricau-Bouvery and Rodolakis, 2005). During abortions in goats and parturition of infected does, a large number of bacteria are excreted by infected animals into the environment (Arricau Bouvery et al., 2003; Roest et al., 2012). Inhalation of pathogen-contaminated aerosols is the main route of infection in humans (Arricau Bouvery et al., 2003). Most of the infected human individuals (~60%) remain asymptomatic after exposure to the pathogen. In symptomatic patients acute infections are usually presented as flu-like illness and pneumonia. Around 1–5% of these cases can develop into chronic infections often leading to life-threatening endocarditis (Maurin and Raoult, 1999; Arricau-Bouvery and Rodolakis, 2005; Raoult et al., 2005; Mazokopakis et al., 2010).

Q fever is prevalent throughout the world, but during 2007–2010 an unprecedented Q fever outbreak occurred in The Netherlands and is referred to as the largest outbreak ever reported (Raoult et al., 2005; Roest et al., 2011b; Hammerl et al., 2015; Kuley et al., 2016). During this epidemic, several isolates from different host species were isolated and cultured. Genotyping by Multiple Locus Variable-number Tandem Repeat Analysis (MLVA) revealed the predominant presence of the so-called CbNL01 genotype and, to a lesser extent, the CbNL12 genotype among the strains isolated from The Netherlands (Roest et al., 2011a). The CbNL01 genotype was identified mostly in strains isolated from goats and specifically from human patients, indicating that goats harboring the CbNL01 genotype strain were most likely the source of the large human Q fever outbreak in The Netherlands. These genotyping studies confirmed the earlier observed epidemiological link between an increase in human Q fever cases and a high abortion rate in goats (Enserink, 2010; van der Hoek et al., 2010; Roest et al., 2011a,b; Kampschreur et al., 2013; Kuley et al., 2016). Additionally, the genotyping studies also showed that the CbNL12 genotype was majorly associated with strains originating from cattle and rarely with strains from goat, sheep and humans (Roest et al., 2011a,b; Tilburg et al., 2012c; Mori et al., 2013).

As an intracellular pathogen, the most important factor for natural selection of *C. burnetii* could be the interactions with its specific host niches. Genome reduction and the presence of mobile genetic elements and virulence related pseudo-genes throughout the genome are predicted to be specific genome manifestations of the obligate intracellular lifestyle of this pathogen (Beare et al., 2009). Hence, a comparison of *C. burnetii* genome sequences with specific emphasize on genes involved in pathogen-host (cell) interactions or modulation thereof, may shed light on adaptation mechanisms of *C. burnetii* to various host species. Currently lipopolysaccharide (LPS) is the only biologically validated virulence factor of *C. burnetii* and relatively little is known regarding other potential virulence factors, or factors involved in pathogen-host interactions and host specificity (Beare et al., 2009; Gilk, 2012; Kuley et al., 2015a). Previous studies showed the up-regulation of several genes in a Dutch outbreak strain (602) under *in vivo* conditions as compared to *in vitro* cell-based and cell-free growth conditions. These studies suggested a role of these up-regulated genes during intracellular survival and replication of *C. burnetii* in hosts. Unfortunately, a large number of the identified *in vivo* up-regulated genes were of unknown function limiting the information derived from this study with regard to adaptation processes of the bacterium in hosts (Kuley et al., 2015a).

*Coxiella burnetii* strains with a different genotypic profile can infect a variable range of host species with a different efficiency. For example; the CbNL01 genotype strains are predominantly found in goats and in humans, whereas the CbNL12 genotype strains are commonly found in cattle and hardly in goats and humans (Roest et al., 2011a; Tilburg et al., 2012b,c; Mori et al., 2013). This suggests a higher susceptibility of humans and goats to *C. burnetii* of the CbNL01 genotype than to the CbNL12 genotype strains. Identification of the genomic variations between different genotypic strains by comparative analysis using bioinformatics approaches may help in the identification of the genetic factors of *C. burnetii* involved in its host and cell tropism. Till now this has not been assessed with the Dutch outbreak strains. Therefore, in the present study, we sequenced and compared whole genome sequences of several strains with different genotypic profiles and originating from various hosts to assess host-specific and/or genotype-specific genetic signatures of *C. burnetii*. Main focus during this study was on strains of the CbNL01 genotype which were predominantly found in The Netherlands during the large outbreak, in order to identify genomic signatures associated with their hyper-virulence behavior. From the performed research, we conclude that an increased genome plasticity and an increased number of single nucleotide polymorphisms in genes, potentially involved in virulence and the evasion of bacterial recognition by the host immune system, is linked to the increased epidemic potential of the Dutch outbreak strains.

## Materials and methods

### Bacterial strains and isolation of genomic DNA

A summary of the features of all the *C. burnetii* strains used in this study is indicated in Table 1. The strains from the Netherlands were primarily isolated from aborted placentas of goats, sheep and from heart valves of human chronic infected patients during the Q fever outbreak period (2007–2010; Roest et al., 2011a). This study also involves strains isolated from aborted placentas of goats and cattle from France. Additionally, a few strains were isolated from Q fever infected humans from elsewhere in the world. All the strains were genotyped based on MLVA (Roest et al., 2011a). The majority of the strains used for sequencing were cultured with a minimal number of sub-passages in BGM cells (Roest et al., 2012). A few strains were cultured axenically in acidified citrate cysteine medium (ACCM-2) also maintaining a minimum number of sub-passages to prevent phase variation (Table 1; Omsland et al., 2011). Genomic DNA was isolated using the phenol-chloroform method (Tang et al., 2013) after overnight incubation with ATL lysis buffer and proteinase K (QIAGEN, Hilden, Germany). A prior DNase treatment was performed to the bacterial pellet of cell-culture cultivated strains to eliminate most of the host derived DNA.

**Table 1:** Summary of *C. burnetii* genome meta-data.

**Strain**	**Genotype**	**Place**	**Origin**	**Media^b^**	**Genome**	**Plasmid**	**Putative CDS**	**GC%**	**Plasmid**	**Accession number**		**Scaffold**	**References**
										**Genome**	**Plasmid**		
NL3262	CbNL01	NL^a^	Goat	CF	2,093,477	37,320	2,101	42.9	QpH1	CP013667	CP013668	–	Kuley et al., 2015b, 2016
CbCVIC1	CbNL01	NL	Goat	CC	1,988,699	37,397	2,138	42.6	QpH1	CP014549	CP014550	NL3262	This study
602	CbNL01	NL	Goat	CC	1,945,399	37,640	2,078	42.6	QpH1	CP014836	CP014837	NL3262	Kuley et al., 2015a,b
NLhu3345937	CbNL01	NL	Human chronic Q fever	CC	1,964,125	37,397	2,110	42.6	QpH1	CP014354	CP014355	NL3262	Kuley et al., 2015b, 2016
42785537	CbNL01	NL	Human chronic Q fever	CF	1,997,221	37,447	2,212	42.6	QpH1	CP014548	CP014547	NL3262	This study
601	CbNL12	NL	Goat	CC	1,963,598	37,407	2,079	42.5	QpH1	CP014551	CP014552	NM	Kuley et al., 2015b
18430	CbNL12	NL	Sheep	CC	1,964,871	37,407	2,078	42.6	QpH1	CP014557	CP014558	NM	This study
701CbB1	CbNL12	France	Cattle	CF	1,966,478	37,457	2,082	42.5	QpH1	CP014553	CP014554	NM	This study
2574	CbNL12	NL	Cattle	CF	1,967,280	37,457	2,081	42.5	QpH1	CP014555	CP014556	NM	This study
Henzerling	Henzerling	Italy	Human acute Q fever	CC	1,956,892	37,394	2,064	42.6	QpH1	CP014559	CP014560	331^d^	Glazunova et al., 2005
Heizberg	Heizberg	Greece	Human acute Q fever	CC	1,956,650	37,394	2,062	42.6	QpH1	CP014561	CP014562	331	Glazunova et al., 2005
Scurry	Scurry	USA	Human chronic Q fever	CC	1,971,034	–^c^	2,020	42.5	–^c^	CP014565	–	Q212^e^	Glazunova et al., 2005
Schperling	Schperling	Kyrgyzstan	Human acute Q fever	CF	2,004,282	37,406	2,087	42.5	QpRS	CP014563	KY271744	Q154^f^	Glazunova et al., 2005

a *Netherlands*.

b *Cell-free (CF) Cell culture (CC) of C. burnetii strains*.

c Plasmid integrated in chromosome

d *CbRSA331*.

e *CbuG_Q212*.

f *CbuK_Q154*.

### Genome sequences of *C. burnetii*

Draft genome sequences of all *C. burnetii* strains (except 602) were *de novo* reconstructed from Illumina MiSeq paired-end 250 bp reads using SPAdes-3.6.2 (Bankevich et al., 2012). The draft genome sequence of the 602 strain was obtained by using Roche 454XL sequencing technology and was *de novo* assembled by Newbler 2.6. The Dutch outbreak representative strain NL3262 was fully reconstructed using a combination of sequencing technologies (Illumina MiSeq paired-end 250 bp, PacBio RS and Roche 454XL) as described previously (Kuley et al., 2016). The contigs of the draft genome sequences were scaffolded and joined into an artificial chromosome based on homology with the closest *C. burnetii* strain from the database using BLAT synteny tool (Table 1; Kent, 2002). Mapping the reads back on the draft genome sequences using Bowtie2 aligner (Langmead and Salzberg, 2012) confirmed its correct synteny and occasionally helped to close minor gaps. All *C. burnetii* (complete and draft) genome sequences were annotated by the NCBI Prokaryotic Genome Annotation Pipeline (http://www.ncbi.nlm.nih.gov/genome/annotation_prok). The (draft) genome sequences and plasmids of all sequenced *C. burnetii* strains have been deposited at GenBank under accession numbers indicated in Table 1. Published genomic sequences of several other *C. burnetii* strains used for phylogenetic studies were obtained from GenBank (Table 2).

**Table 2:** Genome sequences of *C. burnetii* strains obtained from NCBI database for phylogenetic analysis and their *in silico* MLVA genotype.

**Strain**	**Accession no**.	**Source**	***In silico* MLVA**	**Genome (bp)**	**Plasmid type**	**References**
NMRSA493^c^	NC_002971.3	Tick	NM	1,995,281	QpH1	Beare et al., 2009
Cb175^c^	HG825990.3	Human	NM	1,989,565	QpH1	D'Amato et al., 2015
Cb185^d^	NZ_CBTH000000000.1	–	NM	1,991,515	unknown	–
Z3055^c^	PRJEB1438	Sheep	^*^	1,995,463	QpH1	D'Amato et al., 2014
Cb109^d^	AKYP00000000	Human	CbNL01	2,030,000	QpH1	Rouli et al., 2012
EV-Cb_C13^d^	CCAM01000000	Ruminant	CbNL01	2,023,172	unknown	Sidi-Boumedine et al., 2014
NL-Limburg^d^	JZWL00000000	Human	CbNL01	2,214,254	QpH1	Hammerl et al., 2015
Cb_B1^d^	CCAH01000000	Ruminant	CbNL12	2,008,014	QpH1	Sidi-Boumedine et al., 2014
EV-Cb_BK10^d^	CCAL01000000	Ruminant	CbNL12	1,999,727	unknown	Sidi-Boumedine et al., 2014
Cb_B18^d^	CCAI01000000	Ruminant	CbNL12	2,008,445	unknown	Sidi-Boumedine et al., 2014
CbRSA331^c^	NC_010117.1	Human	CbRSA331	2,016,427	QpH1	–
Dugway^c^	NC_009727.1	Rodents	Dugway	2,158,758	QpDG	Beare et al., 2009
CbuG_Q212^c^	NC_011527.1	Human	CbuG_Q212	2,008,870	integrated	Beare et al., 2009
CbuK_Q154^c^	NC_011528.1	Human	CbuK_Q154	2,063,100	QpRS	Beare et al., 2009
Q321^d^	AAYJ01000000	Cattle	Q321	2,004,584	QpDV	Beare et al., 2006
Goat Q177^d^	NZ_AAUP00000000.2	Goat	Goat Q177	2,090,565	QpRS	–
Cb_O184^d^	CCAK01000000	Ruminant	Cb_O184	2,168,222	QpRS	Sidi-Boumedine et al., 2014
Namibia^d^	CP007555	Goat	Namibia	2,101,438	QpRS	Walter et al., 2014a
AuQ01^d^	JPVV00000000	Human	AuQ01	2,073,000	QpRS	Walter et al., 2014b

c *Complete genome sequence*.

d *Draft genome sequence*.

* *In silico MLVA genotype more similar to NM genotype*.

*–Information not available*.

*Integrated, plasmid sequences integrated in chromosome*.

*Unknown, plasmid type not known for these C. burnetii strains*.

### Genotyping of *C. burnetii* strains

All the sequenced strains were genotyped by MLVA using a selection of 10–12 loci described previously (Arricau-Bouvery et al., 2006; Roest et al., 2011a). As an additional verification step, genotypes of the reconstructed genome sequences as well as published genome sequences of strains from NCBI database were checked by *in silico* MLVA genotype analysis using isPCR (BLAT), MLVA primers and genotype references. *In silico* genotypes of all *C. burnetii* sequenced strains corresponded with known/measured MLVA genotypes. The *in silico* analysis of the database strains gave us information on their MLVA genotype (Table 2). Based on the number of repeats per genome loci a minimum spanning tree method was used to cluster the MLVA genotypes using Bionumerics (version 6.6) with default settings.

### Sequence clustering and phylogenetics

Reconstructed as well as database obtained *C. burnetii* sequences were hierarchical clustered based on their MUMi distance (Deloger et al., 2009) and displayed as a phenogram using the BioNJ algorithm (Gascuel, 1997). The underlying MUMi distance matrix was calculated from the pair-wise non-overlapping maximal unique matches (MUMs; using Nucmer version 3.22; Kurtz et al., 2004). Relative pair-wise distances (MUMi) were obtained by dividing the sum of pair-wise MUMs by the average genome size of the two compared genomes. This MUMi distance varies between 0 and 1 where 0 indicates very similar genomes and 1 is for very distant genomes. MUMi trees were visualized in SplitsTree4 (Huson and Bryant, 2006).

For SNP detection in the sequenced draft genomes and in the complete genome of strain NL3262, reads with low quality (bases with a quality score of ≤ Q20) were removed before SNP calling. High quality reads were used to map against reference NM genome (GenBank: NC_002971.3) using Bowtie2 short-read aligner (Langmead and Salzberg, 2012). SNP calling was performed using bcftools (Li et al., 2009) using default parameters. SNP sequence alignments were made for only those positions in NM reference strain where each of the sequenced strain had sequence coverage. Phylogenetic trees based on SNPs were created by Clustal Omega (https://www.ebi.ac.uk/Tools/msa/clustalo/) using standard settings (Sievers et al., 2011) and visualized in SplitsTree4 (Huson and Bryant, 2006). SNPs and insertions/deletions in CbNL01 and CbNL12 strains compared to NM were verified at nucleotide level of the mapped reads.

### Comparison of *C. burnetii* genome contents

Alignment of the complete genome sequence of strain NL3262 with the sequences of the reference NM, and strains which were related to the outbreak CbNL01 genotype (Z3055, NL-Limburg) (D'Amato et al., 2014; Hammerl et al., 2015) was performed by using Mauve 2.4.0 aligner software with default parameters (Darling et al., 2004). Protein orthologs analysis of *C. burnetii* amino acid sequences obtained from GenBank files from NCBI were performed using Proteinortho software (Version 5) with default settings (Lechner et al., 2011). Orthologs and unique proteins in sequenced strains were identified from the orthologous list generated by Proteinortho. Additionally, ortholog analysis of sequenced strains was conducted using the proteome of NM strain as a reference. Pseudo-genes were excluded from the ortholog analysis. Orthologs from different strains were plotted by means of Edwards-Venn diagrams using Venerable package (Version 2.0). For a more detailed genomic sequence comparison, Artemis Comparison Tool (ACT) (Carver et al., 2005) was used to visualize whole genome MUMmer alignments. Detailed analyses were performed on virulence related genes such as variants (mutations/deletions) in LPS encoding region, which were performed by comparing *C. burnetii* genome sequences to LPS encoding region of NM-I (GenBank: AF387640). Also, variants found in the CbNL01 and CbNL12 strains were subsequently pairwise checked against the reference NM strain using ACT and ClustalW (Thompson et al., 1994). We also performed a COG (Cluster of Orthologous Groups) enrichment-analysis on all non-synonymous genes in CbNL01 and CbNL12 strains and analyzed the proportions of different proteins for each COG category.

## Results

### Genome sequences of *C. burnetii* strains

In the present study we report the new genome sequences of 11 *C. burnetii* strains. The reconstruction of the genome sequences of the NL3262 and NLhu3345937 strains was described before (Kuley et al., 2016). Table 1 is a summary of the main features of the sequenced genomes. Except for strain NL3262, all the other strains are reported as draft genome sequences. The assembled contigs of these sequenced strains are joined into artificial chromosomes based on scaffolding against the closest genome sequence present in the database. In *C. burnetii* genome sequences, the insertion sequence (IS) elements are dispersed all over the chromosome and are not found on the plasmid (Seshadri et al., 2003). These elements are repetitive in nature and as a consequence, the majority of contig-breaks in the draft genome assemblies mapped to the positions of an IS sequence, when analyzed against the NM reference genome sequence (GenBank: NC_002971.3). As expected, all *C. burnetii* sequenced genomes possess a single circular chromosome of around 2 Mb and a QpH1 plasmid of 37 kb size (except the Schperling and Scurry strains). The bacterial genomes of all sequenced strains are predicted to contain between 1800 and 2062 coding sequences (CDSs). Around 35–39% of these CDSs were annotated as encoding hypothetical products based on the NCBI Prokaryotic Genome Annotation Pipeline. *In silico* MLVA typing of the genome sequences corresponded with similar profiles of strains as measured by lab-based MLVA measurements (Table 2).

### Genotype-specific structural similarities between *C. burnetii* genome sequences

Whole (complete and draft) genome-based phylogenetic analysis was used to infer the relationship between sequenced *C. burnetii* strains of different genotypes. The strains were initially isolated from different hosts such as goats, sheep, cattle and human patients (both acute and chronically infected; Table 1). The analysis also included whole genome sequences of *C. burnetii* strains that have become available in the NCBI database (Table 2). Genome sequences of some of the strains from the database, present as draft sequences (several contigs), were scaffolded similarly to our reconstructed genomes and joined into artificial chromosomes based on the reference NM strain. The phylogenetic analysis of these genome sequences were based on MUMi distance. This approach takes into account the number of maximum unique and exact matches (MUMs) of a given minimal length shared by the two genome sequences being compared (Kurtz et al., 2004; Deloger et al., 2009). The phylogenetic analysis was conducted for genomes excluding the plasmids, as presence or absence of plasmids did not result in any difference in hierarchical clustering of *C. burnetii* strains.

**Figure 1:**
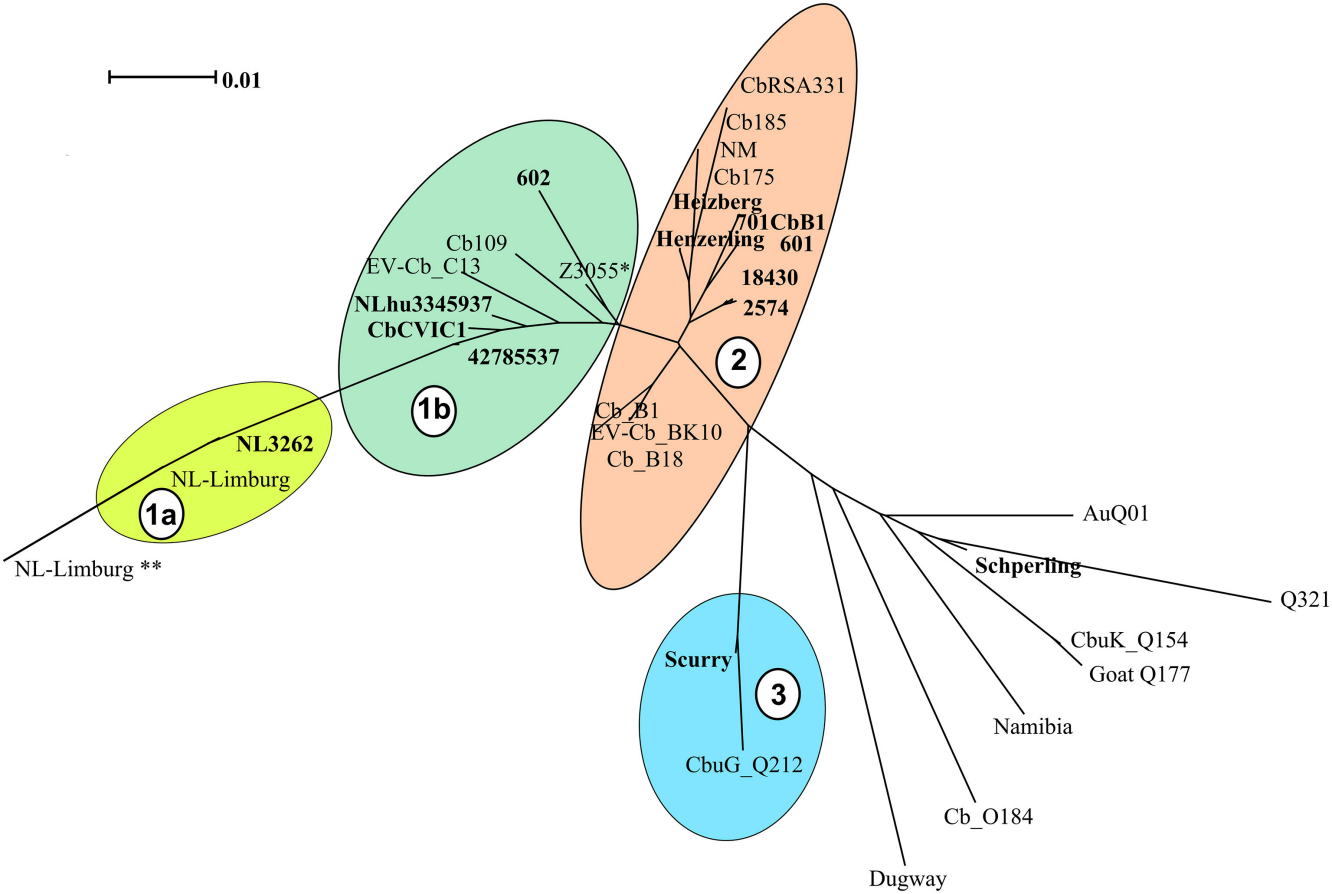
Phylogenetic relationships among *C. burnetii* genome sequences. *C. burnetii* whole (complete and draft) genomes from newly sequenced strains and strains obtained from NCBI database were hierarchical clustered and are displayed as a phenogram using the BioNJ algorithm. Strains in bold font are newly sequenced strains and other strains are genome sequences obtained from NCBI database. Scale bar indicates 1% of genome variability. Clades representing strains from the same MLVA genotype are indicated by colored clouds. Clades contain strains with distance ranging from 0.010 to 0.021. Clade 1a and 1b: CbNL01, Clade 2: CbNL12 and NM like genotype, Clade 3: Scurry genotype (Plasmid-less strains), non-clustered strains are each of different genotypes (except for the sequenced Schperling strain and NCBI obtained Q321 strain, which are of same genotype). ^*^Represents the genome of strain Z3055 which is equidistant from cluster 1 and 2, ^**^ represent the genome of strain NL-Limburg with duplicated regions.

**Figure 2:**
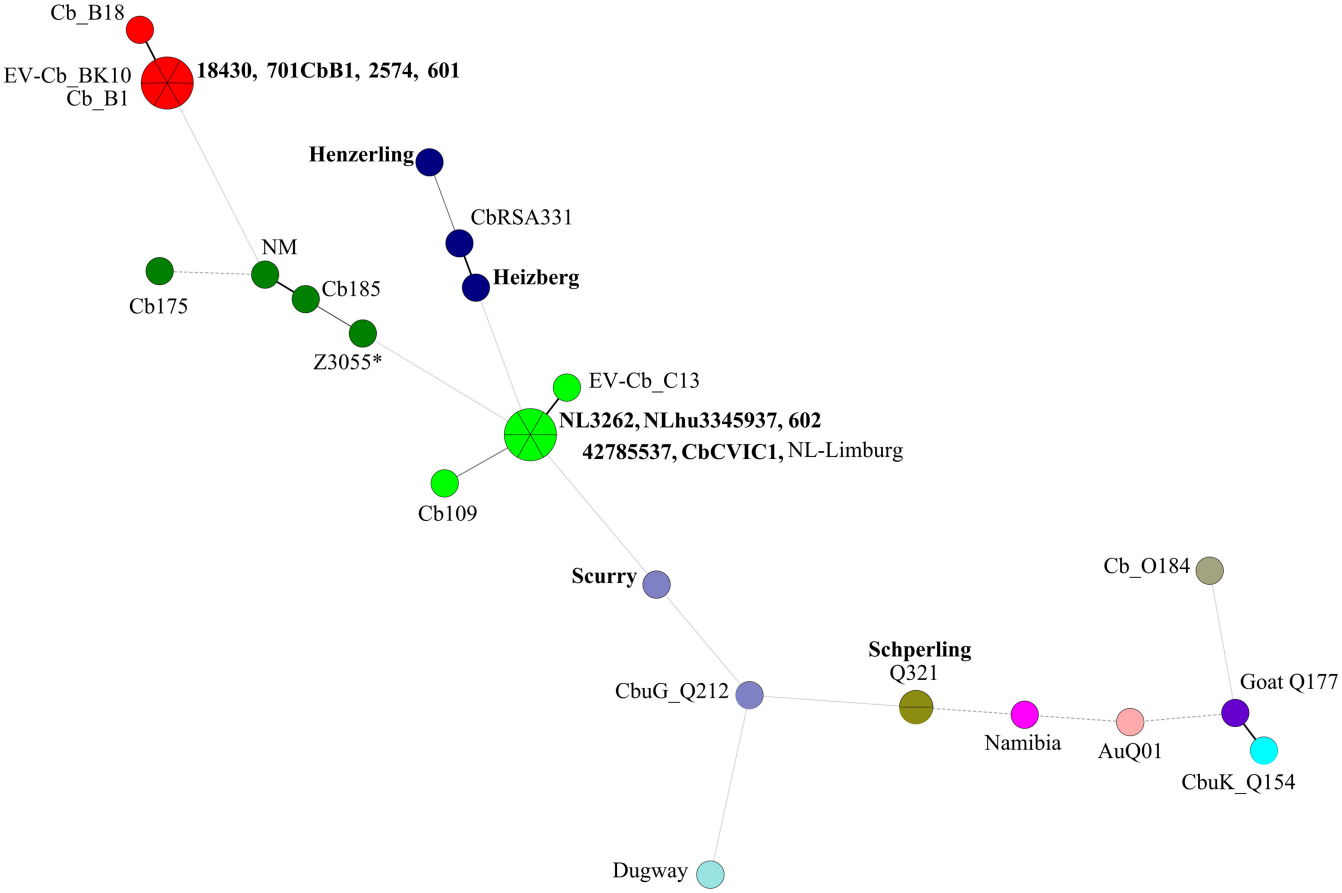
Minimum spanning tree (MST) analysis of the *in silico* MLVA genotypes from *C. burnetii* strains used in this study and sequences obtained from NCBI database. A total of 12–17 micro satellite loci (depending on coverage in each particular genome sequence) were used to construct a dendogram using Bionumerics (version 6.6). Each circle represents a strain and the size of the circle corresponds to the number of strains with the same genotype. Colors indicate the genotype of the strain. The connecting lines refer to the distance, while the intensity of lines shows closeness between the strains. Strains in bold font are newly sequenced strains and other strains are sequences obtained from NCBI database. ^*^Represents the *in silico* MLVA genotype of strain Z3055 (the majority of the MLVA markers are of the NM genotype and a few of the CbNL01 genotype).

Based on the clustering analysis, the strains could be divided into 4 main clades (indicated as 1a, 1b, 2 and 3 in Figure 1). The majority of the *C. burnetii* sequenced strains segregated into two clusters [cluster 1 (1a and 1b) and cluster 2, Figure 1]. These two clusters correspond to the two major genotypes (CbNL01 and CbNL12) of strains isolated from The Netherlands, respectively. Strain NL3262 and the database strain NL-Limburg clustered closely together (Cluster 1a, Figure 1). The genome of strain NL3262 is a complete reconstructed genome derived and curated using different sequencing technologies (Kuley et al., 2016). The NL-Limburg genome is a draft genome generated with a single sequencing technology (PacBio) and its assembly constitutes 4 contigs representing the chromosome (Hammerl et al., 2015). The contigs of NL-Limburg cover the complete genome of NL3262 without any gaps (as visualized by ACT; data not shown). The ends of the contigs in NL-Limburg contain overlapping sequences of around 50,000 bp. Due to these additional sequences (which are probably the result of assembly error) the genome of strain NL-Limburg (represented as ^**^ in Figure 1) is clustered at some distance away from strain NL3262 (MUMi distance 0.025). Upon removal of these repetitive sequences the MUMi distance between strains NL-Limburg (indicated in cluster 1a without ^**^) and NL3262 decreases to 0.01, indicating nearly identical genomes with differences based on 8 SNPs. Draft genome sequences of the database strains (Cb109 and EV-Cb_C13) belonging to the CbNL01 genotype (Figure 2 and Table 2) clustered with the CbNL01 strains (602, CbCVIC1, 3345937, 42785537) originating from The Netherlands in cluster 1b (Figure 1). Strains NL3262 and NL-Limburg belong to the CbNL01 genotype strains as well (Figure 2 and Table 2; Hammerl et al., 2015; Kuley et al., 2016). The major differences between the complete and draft genome sequences of the strains belonging to the clusters 1a and 1b are the genomic locations of highly repetitive transposon sequences. The genome sequences of strains NL3262 and NL-Limburg contain 121 and 128 IS elements respectively scattered around the chromosome. These IS elements are missing in draft genome sequences, most probably due to their presence at contig-break regions, as indicated above. Thus, due to these structural differences, the draft genome sequences cluster very closely to each other (Cluster 1b) relative to the complete genome sequences (Cluster 1a). The complete sequence of NL3262 and the nearly complete genome sequence of NL-Limburg (belonging to genotype CbNL01) differ with a MUMi distance of around 0.05–0.06 from the draft genome sequences in cluster 1b which also belong to CbNL01 genotype.

Cluster 2 includes the draft genome sequences of strains Henzerling, Heizberg and the sequences of the CbNL12 genotype group of strains 601, 701CbB1, 2574, and 18430 (Figure 1). Apart from these, cluster 2 contains the database strains belonging to the CbNL12 genotype (draft genome sequences: CbB1, Cb_B18, and EV-Cb_BK10) as well as the reference NM like genotype strains (NM, CbRSA331, Cb175, and Cb185) (Figure 2 and Table 2). The complete genome sequence of strain Z3055 was placed at an equidistance (MUMi distance 0.02) from clusters 1 (CbNL01) and 2 (CbNL12) (Figure 1). An *in silico* PCR analysis of strain Z3055 showed few MLVA markers similar to CbNL01 genotype but the majority indicative for the NM genotype. This resulted in the presence of Z3055 in-between the nodes of the NM and CbNL01 genotype strains in an MST analysis (Figure 2). Based on the MST and phylogenome analysis, we assessed the genome sequences of strains Z3055 and NM to be very closely related. Cluster 3 included strain Scurry and the database strain CbuG_Q212, both belonging to the same genotype (Figures 1, 2). These strains clustered separately as they contain plasmid-homologous regions integrated in their genomes unlike other strains (Figure 1 and Table 1; Willems et al., 1997). From the phylogenetic analysis, the Schperling strain was found to be the most distant strain as compared to the other *de novo* sequenced strains used in this study (MUMi distance 0.05; Figure 1). The genome sequences of strains Q321, CbuK_Q154, and Goat Q177 were most similar to the strain Schperling with a MUMi distance of around 0.025–0.03. However, except for strain Q321, these strains belonged to different genotypes (Figure 2). Hence, a phylogenetic clustering of the strains based on an MLVA classification rather than on the host-origin background indicated a high-level of genome sequence similarity of genotype-specific *C. burnetii* strains.

**Figure 3:**
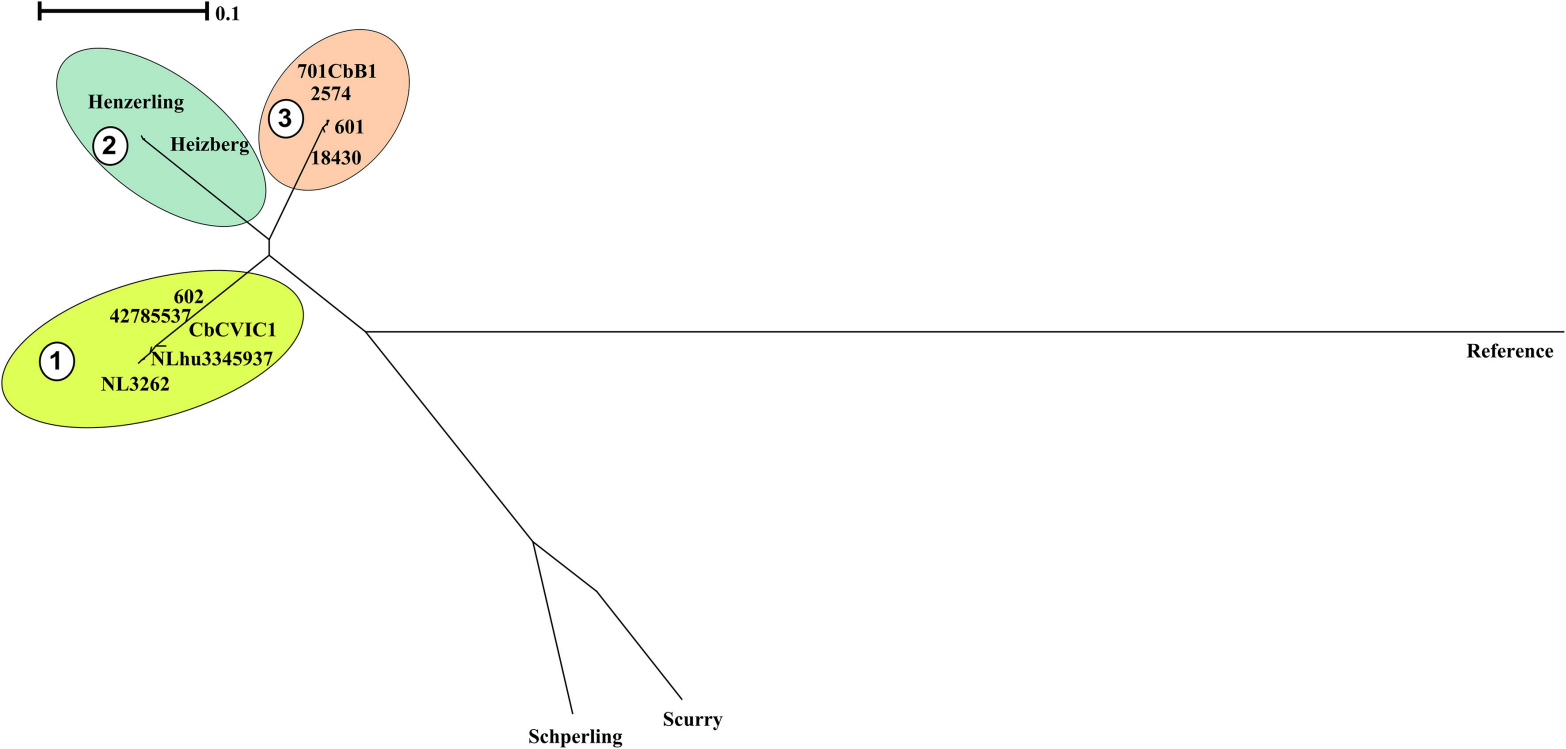
Phylogenetic relationships among sequenced *C. burnetii* genomes based on SNP analysis. The phylogenetic tree is based on SNPs among genomes of the sequenced strains with respect to the genome of reference strain NM using bcf tools. The SNP tree was constructed using Clustal Omega and the clustering is displayed as a phenogram using the BioNJ algorithm. The scale bar indicates the number of substitutions per site. Clades representing strains from the same genotype are indicated by colored clouds. Clade 1:CbNL01, Clade 2: Henzerling and Heizberg (similar genotype), Clade 3: CbNL12. The Schperling and Scurry strains are present separately due to the presence of distinct SNPs relative to other sequenced strain genomes. NM indicates the polymorphic regions in sequenced strain genomes relative to the NM reference genome.

A phylogenetic tree was also reconstructed using the identified SNPs among the sequenced strains with respect to the reference strain NM using bcftools (Figure 3; Li et al., 2009). The SNP tree was constructed using Clustal Omega (Sievers et al., 2011). At SNP level, strains belonging to similar genotypes clustered together with clade 1 containing the CbNL01 genotype strains; clade 2 containing the strains Henzerling and Heizberg and clade 3 containing the CbNL12 genotype strains. This indicates that the presence of similar SNPs in the genomes of the same genotype strains relative to the reference strain NM. Strains Schperling and Scurry clustered separately, indicating that these strains contain SNPs distinct from the SNPs present in other sequenced strains compared to the reference strain NM. The data corresponds to the presence of distinct genotypes of Schperling and Scurry compared to other sequenced strains (Figure 2). This SNP based clustering of genotype-specific strains further confirms the highly similar genome sequences of strains belonging to the same genotype.

### Conserved and unique gene content in *C. burnetii* sequenced genomes

A comparison of the genome sequences of *C. burnetii* shows that all sequenced strains are highly conserved in terms of size, number of coding sequences and in nucleotide composition (Table 1). The degree of conservation is illustrated by the comparison of the coding capacity of these genomes: 1447 predicted proteins are orthologs in all sequenced 13 *C. burnetii* strains in this study (with a total CDS count ranging from 1800 to 2062) among which 1409 of the predicted proteins are orthologs with reference NM. Remaining 38 proteins not shared with NM are majorly hypothetical protein products mostly occurring due to modifications (such as point mutations, small deletions/ insertions of <50 bp) in existing NM pseudo-genes (Supplementary Table 1). The 1409 orthologs among all strains constitute the coding core genome. All sequenced *C. burnetii* strains contain the QpH1 plasmids, except for strain Schperling containing QpRS plasmid and the plasmidless strain Scurry. The QpH1 plasmids from sequenced strains were all very similar to the reference NM QpH1 plasmid (Accession No. NC_002118.1) with differences on an average of 58 point mutations. The draft plasmid sequence of Schperling was similar to the published sequence of plasmid QpRS (Accession No. Y15898.1) with differences in 63 SNP and a gap of 1810 bp (data not shown).

Ortholog analysis of the proteome of the sequenced *C. burnetii* strains showed the presence of a few unique proteins annotated as hypothetical protein in CbNL01 and CbNL12 strains (data not shown). Further analysis of genes encoding these unique proteins showed that these genes were present at the beginning or end of contigs in assembled draft genome sequences. As contig ends of draft genome sequences are highly repetitive, genes annotated in these regions were not considered as unique genes, because these genes were truncated transposase genes. Taking this factor into account and other manual annotation corrections, we found no unique complete genes encoded in CbNL01, CbNL12, Henzerling, and Heizberg strains. Instead of unique genes for each strain we did find orthologs that were genotype-specific. These orthologs were shared between strains of the same genotype only and were annotated as hypothetical proteins. When we compared these genotype-specific genes at the nucleotide level to the genome of the reference strain NM, mutations or partial ORF deletion in existing NM pseudo-genes were observed for which altered functionality or regulation cannot be directly inferred (Supplementary Table 2).

By comparing the proteome of the 13 sequenced strains (Table 1) 50 and 98 unique protein encoding genes were identified in strains Scurry and Schperling, respectively. Out of these, 3 and 17 genes are exclusively found in Scurry and Schperling, whereas the other genes were orthologs with the closely related strains CbuG_Q212 and CbuK_Q154, respectively (Figure 1). These unique genes in Scurry and Schperling strains were annotated as hypothetical proteins and result from point mutations in existing pseudo-genes of CbuG_Q212 and CbuG_Q154 (data not shown).

### Highly similar *C. burnetii* gene content within the same genotype-groups

**Figure 4:**
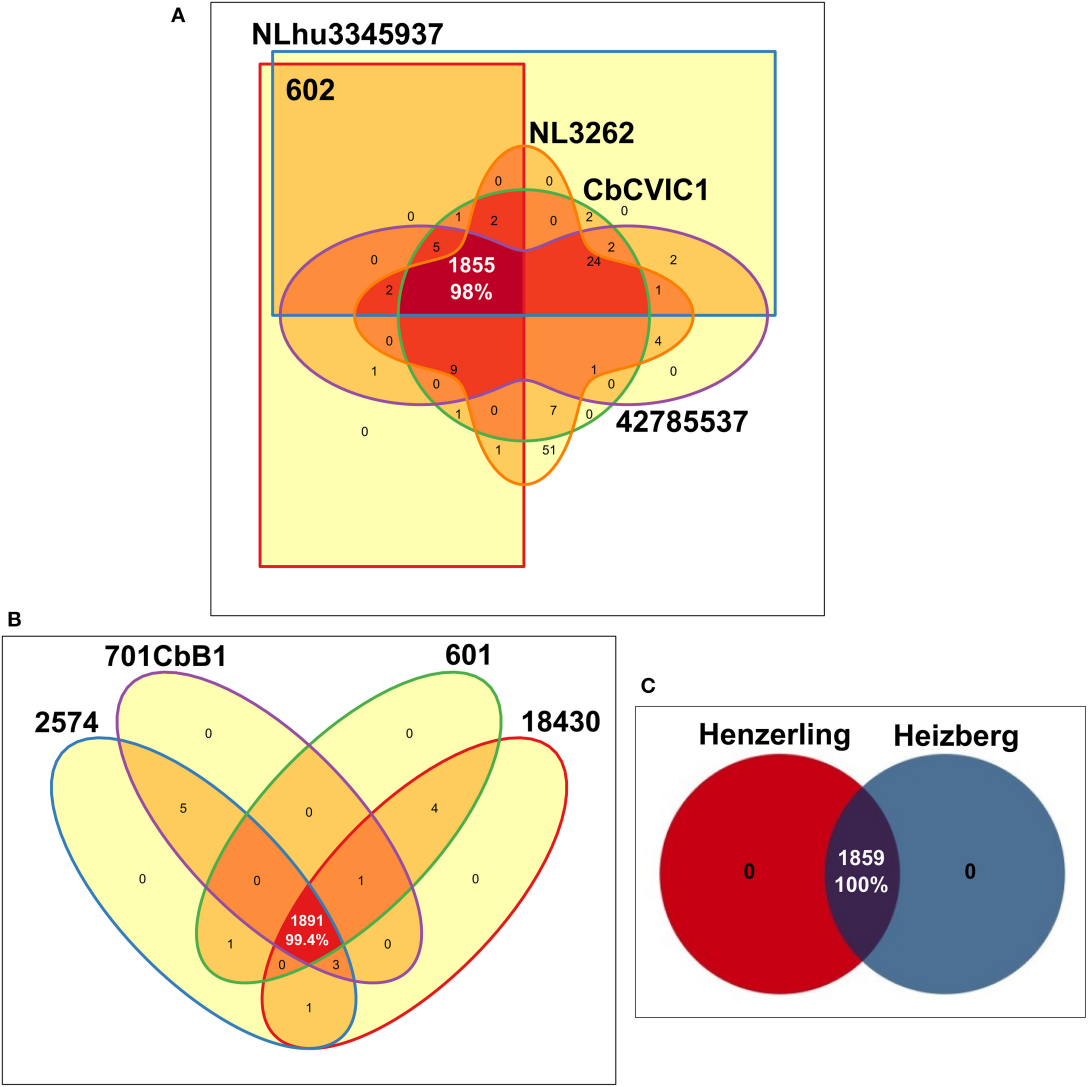
Edwards-Venn diagrams showing the number of orthologs and unique predicted coding proteins among strains of the same MLVA genotype. Number of ortholog genes shared between **(A)** CbNL01 genotype strains **(B)** CbNL12 genotype strains and **(C)** human strains Henzerling and Heizberg is represented by Edwards-Venn diagrams. Transposase coding proteins were included in the analysis and pseudogenes were scored as absent.

A comparison of the coding capacity of the genomes of strains belonging to the same genotype showed 98, 99.4 and 100% orthologs in predicted proteins of CbNL01, CbNL12, and the human strains (same genotype Henzerling and Heizberg strains), respectively (Figure 4). Fifty-one unique genes (non-orthologs) of NL3262 encode for transposase associated proteins (Figure 4A). These genes are not properly annotated in 602, CbCVIC1, NLhu3345937, and 42785537, because they fall within contig-break regions of the draft genome sequences. The high level of similarities of the proteomes between these strains indicates that the gene content is highly conserved within strains of the same genotype. As all the 13 *C. burnetii* strains included in this study were initially isolated from different hosts, a comparison was made to assess the percentage of proteins shared by strains derived from each host-species. These comparisons resulted in the presence of 94.6, 100, 87.9, and 87% orthologs in predicted proteins of goat, cattle, human-acute, and human-chronic isolated strains respectively (Supplementary Figure 1). The lower percentage of orthologs shared by strains obtained from various host species is due to the presence of different genotypes within each host-species, except for the cattle-derived strains, as both cattle strains were of same CbNL12 genotype. For example; the strains isolated from goat include both CbNL01 (NL3262, 602, CbCVIC1) and CbNL12 (601) genotypes (Supplementary Figure 1B). Further, the strains isolated from acute and chronic human Q fever patients contain more divergent strains (Schperling and Scurry respectively). Because of this, the protein orthologs of acute and chronic human strains are comparatively less (~87%, Supplementary Figures 1C,D). Taken together, the percentage of orthologs in strains obtained from similar host-species is less conserved than the strains obtained from similar genotype. This clearly indicates the presence of genotype-specific functional characteristics of *C. burnetii* strains.

### Architecture of the NL3262 complete genome sequence

**Figure 5:**
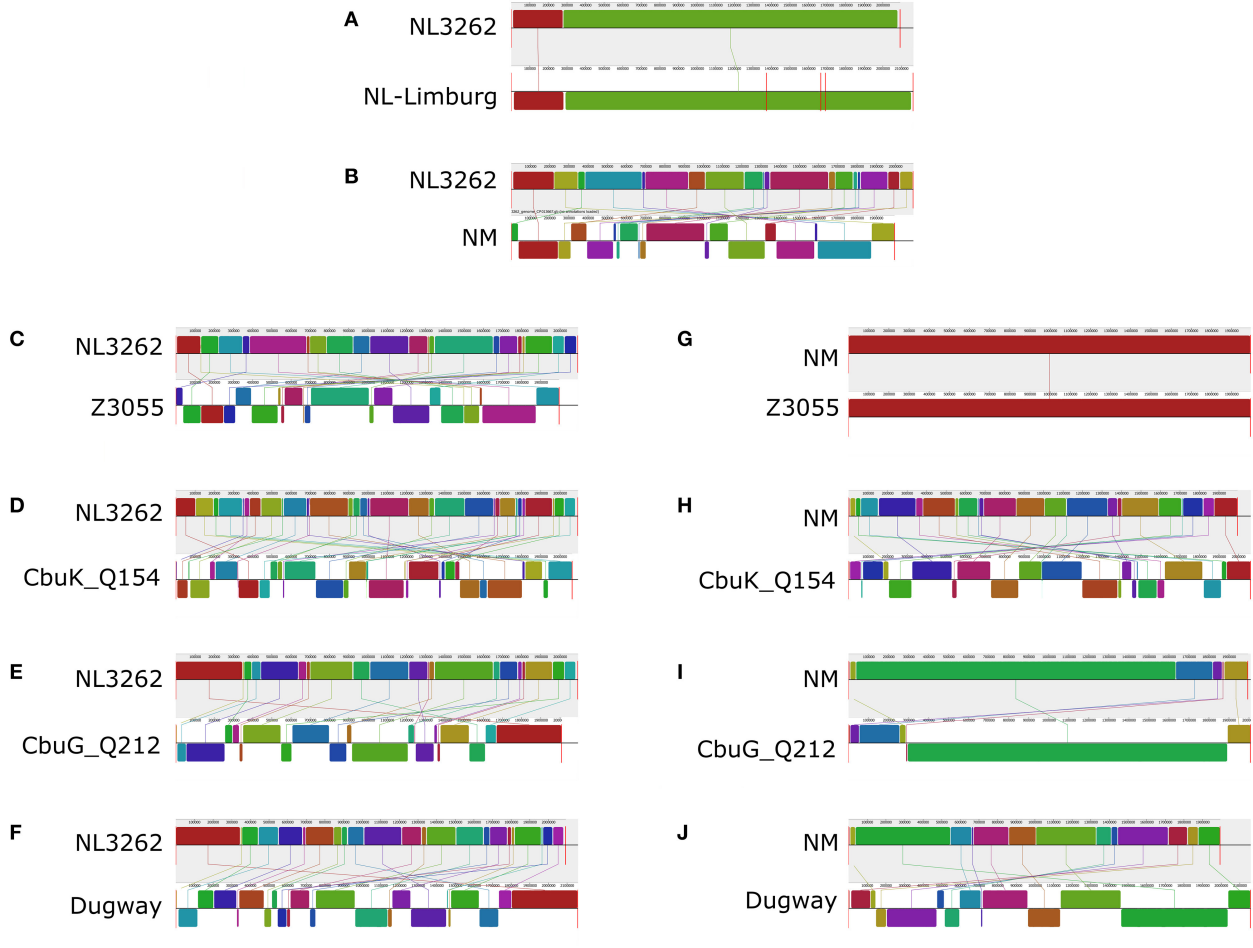
Alignment of the genome sequences of *C. burnetii* strains relative to NL3262 and NM. Comparisons were performed using the Mauve genome alignment tool. The genome rearrangements of strains NL-Limburg **(A)**, NM **(B)**, Z3055 **(C)**, CbuK_Q154 **(D)**, CbuG_Q212 **(E)**, and Dugway **(F)** are depicted with respect to strain NL3262. Genome rearrangements of strains Z3055 **(G)**, CbuK_Q154 **(H)**, CbuG_Q212 **(I)**, and Dugway **(J)** are depicted with respect to strain NM. Vertical red lines for each genome indicate contig boundaries (draft sequences). Color-coded LCBs (Locally Collinear Blocks, regions within genome sequence that are conserved and predicted to be free of any internal rearrangements) indicate conserved segments. Upper and lower LCBs of the genomes correspond toforward and reverse orientation with respect to NL3262. Connector lines indicate orthologous LCBs.

To assess the genome architecture of strain NL3262 and to study its putative genome rearrangements, the complete genome sequence of strain NL3262 was aligned with the genome sequence of strains NL-Limburg, NM, Z3055, Cbuk_Q154, CbuG_Q212 and Dugway using the Mauve genome aligner (Figure 5). Among the different *C. burnetii* strains, the CbNL01 genotype strains NL3262 and NL-Limburg encode the highest number of transposase genes (106-112 IS110 family transposase, 3 ISAs1 family transposase and 12-13 remnant transposase genes, respectively). Other completely sequenced *C. burnetii* genomes generally contain only 30-60 transposase genes (Beare et al., 2009; D'Amato et al., 2014). Compared to the genome sequence of NL3262, chromosomal rearrangements in strains NL-Limburg, NM, Z3055, Cbuk_Q154, CbuG_Q212, and Dugway strains have resulted in 2, 19, 21, 32, 21, and 23 Locally Collinear Blocks (LCBs) of the same gene content and gene order respectively (Figures 5A–F). NM and Z3055 strains share almost all LCBs indicating a similar genome structure (Supplementary Table 3). Cumulatively, the LCBs of NM, Z3055, Cbuk_Q154, CbuG_Q212, and Dugway represent 116 genomic breakpoints relative to the genome of strain NL3262 (Figures 5B–F). In the genome sequence of strain NL3262, 102 (88%) of the breakpoints occurred at intact or a remnant transposase sequences. These transposase mostly belonged to IS110 family and very rarely to IS30 and ISAs1 family (Supplementary Table 3). The presence of transposase genes at almost all genome breakpoints suggests an important role of homologous recombination in order to establish the observed genome rearrangements of the Dutch outbreak strains. Unlike other strains, the gene order in strains NL3262 and NL-Limburg are nearly identical, yielding only 2 LCBs. The absence of rearrangements between NL3262 and NL-Limburg, indicates a similar genome structure of these CbNL01 genotype Dutch outbreak strains isolated from goat and human patient (Figure 5A).

### Increased number of transposons in dutch outbreak CbNL01 MLVA genotype strains

Genome sequencing of a number of Dutch outbreak strains belonging to the CbNL01 MLVA genotype resulted in 132, 135, and 123 contigs for strains NLhu3345937, 42785537, and CbCVIC1, respectively. In contrast, sequencing of the CbNL12 strains 601, 2574, 18430, and 701CbB1 resulted in 43, 40, 66, and 42 contigs, respectively. As described above, the majority of the contig-break points contained repeat sequences and corresponded mostly to transposase genes when mapped against the NM reference genome. Based on this observation, we anticipate that the observed number of contigs roughly corresponds to the number of transposase genes encoded by the strain. Based on this assumption, the CbNL01 and CbNL12 strains contain around 130 and 47 contigs, respectively. The assumed high numbers of transposons in the draft genome sequences of CbNL01 strains also matches with the number of annotated transposase genes in strains NL3262 (121 intact and remnant transposons) and NL-Limburg strains (128 intact and remnant transposons) belonging to the same genotype group. Taken together, the largest number of transposons are encoded by the CbNL01 Dutch outbreak strains as these strains contain two to three fold more transposase genes compared to other strains of *C. burnetii* sequenced so far (Beare et al., 2009; D'Amato et al., 2014).

### Differences in dutch strains at SNP level

To further asses the differences in the genome sequences of the Dutch strains from different genotypic groups, we performed a detailed NM reference-centric variant analysis. For this analysis we used the complete genome sequences of the NL3262 strain (Kuley et al., 2016) and the draft genome sequence of the CbNL01 and CbNL12 strains. We found an average of 2514 SNPs between the CbNL01 strains and NM. Among these, 417 SNPs were present in intergenic regions, 1041 were synonymous SNPs in coding regions and 1056 were non-synonymous SNP which corresponded to 702 mutated genes. Between the CbNL12 strains and NM an average of 2078 SNPs were found, with 340 SNPs in intergenic regions, 889 SNPs in coding regions (synonymous SNP) and 849 non-synonymous SNPs which corresponded to 581 mutated genes. With respect to NM, these mutated genes in CbNL01 and CbNL12 genotype strains majorly encoded hypothetical proteins, membrane proteins, transporter proteins, DNA repair proteins, translation-related proteins and a few virulence-associated proteins (explained in detail below). Compared to the genome of NM, the genomes of the CbNL01 strains showed a deletion of 3,600 bp (containing ankyrin-repeat containing protein; CBU_0072), a deletion of 2,300 bp (containing several hypothetical proteins; CBU_0877-CBU_0880) and a larger deletion of 9,700 bp (containing the peptidoglycan catabolism proteins; CBU_1101-CBU_1112). A deletion of around 2,250 bp (containing several hypothetical proteins; CBU_0016-CBU_0019) was observed in the CbNL01 and CbNL12 genome sequences, relative to the NM genome sequence (Supplementary Figure 2).

With respect to mutations in membrane protein encoding genes, a total of 64 and 48 genes were mutated in the CbNL01 and CbNL12 strains out of the 104 membrane protein encoding genes in the NM strain. Thirty eight of these genes contained the same mutation in both genotype strains. Additional mutations in 26 hypothetical membrane proteins were specific for all the CbNL01 strains. Although, the exact functions of these hypothetical protein products are not known, changes in these cell surface proteins could potentially contribute to an altered antigenic profile of the CbNL01 strains. Large numbers of mutations were also seen in transporter genes, where a total of 41 and 25 genes were mutated in the CbNL01 and CbNL12 strains (from a total of 75 transporters in NM). Among these, 20 genes contained identical mutations in both genotype strains, whereas 21 genes contained mutations specific for the CbNL01 strains. These transporter genes belonged mostly to the ABC (ATP-binding cassette transporters) and MFS (major facilitator superfamily) family transporters. Further COG analysis of the mutated transporter genes showed that these genes could be primarily involved in transportation of amino acid, carbohydrate and ions (COG categories E, G, and P respectively). Moreover, we also identified mutations in several translation-related proteins in the outbreak CbNL01 strains. Around 29 of these mutated genes were also found in the CbNL12 strains, whereas 15 mutated genes were specifically found in the CbNL01 strains (from a total of 121 translation genes in NM; Supplementary Figure 2).

Recognized virulence factors of *C. burnetii* are genes involved in primary defense mechanisms against oxidative stress. These include several DNA repair genes involved in base excision repair, nucleotide excision repair, mismatch repair, addAB mediated recombinational repair systems, and oxidative stress enzymes (Mertens and Samuel, 2012). Our variant analysis showed high number of mutations in the DNA repair genes; wherein 13 mutated genes were shared in both genotype strains and mutations in 7 genes were specific to the CbNL01 strains (from a total of 26 DNA repair genes in NM). On the contrary, we did not find any mutations in genes encoding for oxidative stress enzymes among the various strains indicating that these genes are highly conserved. The secondary defense mechanisms of *C. burnetii* includes the manipulation of host cell processes (Mertens and Samuel, 2012). A Dot/Icm type IV secretion system (T4SS) is predicted to be encoded by the genome of *C. burnetii* homologous to the *L. pneumophila* Dot/Icm system. This is an important transfer system associated with the delivery of effector proteins from the bacterial cell to the host cytosol mediating PV formation and other cellular events required for bacterial maintenance and survival (Seshadri et al., 2003; van Schaik et al., 2013; Kuley et al., 2015a). Among the T4SS genes, 4 genes were mutated in both genotype strains and an additional 4 genes were mutated specifically in the CbNL01 strains. Furthermore, 12 effector proteins characterized by the presence of eukaryotic like protein domains were also mutated in the CbNL01 and CbNL12 strains relative to NM. These genes are predicted to be virulence-related as the encoded proteins contain eukaryotic-like domains which may mimic host proteins and module host responses required for successful bacterial infection (Supplementary Figure 2). Taken together, compared to the reference strain NM, genotype-specific mutations of genes were present in the Dutch strains. Furthermore, a relatively larger number of non-synonymous mutations were present in critical genes of the CbNL01 strains compared to the CbNL12 strains.

### Changes in LPS encoding genes and its effect on phase variation

Repeated *in vitro* passages of *C. burnetii* strains have been shown to induce antigenic variation and loss of virulence characteristics due to the transition into a truncated LPS structure. This truncation of LPS is associated with chromosomal deletions of O-antigen coding genes, located in a 38 kb region in the genome resulting in antigenic phase variation from phase I to phase II (Hoover et al., 2002; Denison et al., 2007; Kuley et al., 2015b). The genome sequences established in this study, were compared with the LPS encoding region (CBU0676–CBU0706) of NM-phase I (GenBank: AF387640) to visualize deletions in the LPS encoding region. The human strains Henzerling, Heizberg, Schperling, and Scurry contained no deletions in this region (Table 3). However, a deletion of 201 bp (NM-phase I LPS encoding region: 14604-14805) was consistently observed in this region in all sequenced (601, 2574, 701CbB1, 18430) and database strains (CbB1, CbB18, EV-Cb_BK10) of the CbNL12 genotype (Table 3). This deletion corresponded to a portion of CBU_0686 gene (old gene name: JB153-5), a paralog predicted to encode a 2-Oxoacid dehydrogenase (Hoover et al., 2002). As a paralogous gene, deletion of a portion of this gene might not affect the LPS synthesis as the function might be compensated by the other gene in the genome. Similarly, a deletion of 354 bp (NM-phase I LPS encoding region: 20476-20830) was consistently observed in this region in all sequenced (NL3262, 602, CbCVIC1, NLhu3345937, 42785537) and database strains (NL-Limburg, Cb109, EV-Cb_C13) of the CbNL01 genotype. This deletion corresponded to a portion of CBU_0691 gene (old gene name: JB153-10, Table 3) and is predicted to be involved in virenose synthesis which is one of the unusual sugars only present in the *C. burnetii* LPS-phase I structure (Toman et al., 1998). Due to the deletion, CBU_0691 gene is frame-shifted and might consequently not encode for enzymes involved in virenose synthesis. We hypothesize that this deletion of 355 bp may induce the start of phase shifting in CbNL01 strains. Since the genome sequences of 602 (CbNL01 genotype) was obtained from *in vitro* grown cell cultures, we looked back into our previous functional analysis data of the 602 strain cultured in cells maintaining a low passage number. Our transcriptome measurements showed that the CBU_0691 gene was actively transcribed in cells (Kuley et al., 2015b). Although the deletion of a portion of this gene results in frame shifting of CBU_0691, it is still possible that gene CBU_0691 is transcribed but that no protein product is formed. In addition, a complete gene deletion (corresponding to CBU_0677 to CBU_0682) and a deletion of part of the genes (corresponding to CBU_0676 and CBU_0683) were observed in the NLhu3345937 strain of the CbNL01 genotype. The NLhu3345937 strain was passaged in cells for only 11 times and already showed deletion of several O-antigen genes, indicating a high rate of phase shifting in this strain under *in vitro* culture conditions (Table 3). Apart from these deletions, non-synonymous mutations in CBU_0688 and CBU_0698 were present in all CbNL01 strains relative to NM. Additionally, mutations in 12 O-antigen genes were observed in all CbNL01 and CbNL12 strains, compared to NM (Supplementary Figure 2). Taken together, we observed genotype-specific deletions and mutations in the LPS encoding region at a higher rate in CbNL01 strains than in CbNL12 strains.

**Table 3:** Deletion of LPS O-antigen encoding genes of *C. burnetii* strains compared to NM-phase I sequence (GenBank: AF387640).

**Strain**	**Genotype**	**Deletion**	**No. of bp**	**Gene containing deletion**	**Remarks**
NL3262	CbNL01	20,476–20,830	354	CBU_0691	Partly^b^
CbCVIC1	CbNL01	20,476–20,830	354	CBU_0691	Partly
602	CbNL01	20,476–20,830	354	CBU_0691	Partly
42785537	CbNL01	20,476–20,830	354	CBU_0691	Partly
NLhu3345937	CbNL01	20,476–20,830	354	CBU_0691	Partly
NL-Limburg^a^	CbNL01	561–7,902	7341	CBU_0676, CBU_0683	Partly
				CBU_0677 to CBU_0682	Full^c^
		20,476–20,830	354	CBU_0691	Partly
Cb109^a^	CbNL01	20,476–20,830	354	CBU_0691	Partly
EV-Cb_C13^a^	CbNL01	20,476–20,830	354	CBU_0691	Partly
601	CbNL12	14,604–14,805	201	CBU_0686	Partly
18430	CbNL12	14,604–14,805	201	CBU_0686	Partly
701CbB1	CbNL12	14,604–14,805	201	CBU_0686	Partly
2574	CbNL12	14,604–14,805	201	CBU_0686	Partly
CbB1^a^	CbNL12	14,604–14,805	201	CBU_0686	Partly
CbB18^a^	CbNL12	14,604–14,805	201	CBU_0686	Partly
EV-Cb_BK10^a^	CbNL12	14,604–14,805	201	CBU_0686	Partly
Henzerling	Henzerling	None	–	–	–
Heizberg	Heizberg	None	–	–	–
Scurry	Scurry	None	–	–	–
Schperling	Schperling	None	–	–	–

a *Database strains*.

b *Deletions of a portion of gene*.

c *Deletions of complete genes*.

## Discussion

In this study we used genome comparisons to detail the extent of genomic diversity of several *C. burnetii* strains. Focus was given to goat and human strains of the Dutch predominant CbNL01 genotype to identify genomic aspects resulting in its anticipated increased virulence characteristics. In all the strains, we firstly performed phylogenome analysis to infer the relationships between the sequenced strains. Further, we analyzed orthologs and mutations among all sequenced strains with respect to the NM reference strain to assess differences between strains at the gene and nucleotide level.

Regardless of whether the sequence data was derived from complete or draft genome sequences, the phylogenetic clustering of the strains was based on the MLVA genotype rather than on host origin. Clustering of the genome sequences was similar to the clustering based on *in silico* MLVA genotyping. Moreover, a similar genotype-specific clustering of strains was observed based on SNP analysis. The inferred topology of *C. burnetii* strains is consistent with different analysis methods and also with previous phylogenetic studies based on MLVA and MST genotypic data (Roest et al., 2011a; Tilburg et al., 2012b,c). Based on these findings, a close relationship of genotype-specific *C. burnetii* strains was clearly observed. The phylogenetic analysis, as described here, is an improved genotyping tool, besides the MLVA and MST methods, as it uses whole genome sequences for comparisons of strains rather than a few genomic loci. Nevertheless, similar results were obtained with the three techniques. Thus the results of our study validate the traditional genotyping methodologies as sensitive and less-time consuming tools used for outbreak investigations and molecular epidemiology of pathogens.

Among the different Dutch outbreak strains, the NL3262 strain has now a complete genome sequence. The NL-Limburg database strain has a draft genome sequence, although with contigs covering the complete genome of NL3262. Both strains are clonal as the difference between these strains is limited to 8 point mutations. Compared to each other, both strains NL3262 and NL-Limburg have only one DNA rearrangement in their genomes (Figure 5) and contain around 121-130 transposase encoding genes. This is the highest number of transposase genes encoded by any other *C. burnetii* strain (Beare et al., 2009). Upon phylogenetic genome analysis, strains NL3262 and NL-Limburg cluster together in cluster 1a. These strains differ from other CbNL01 strains in cluster 1b by point mutations (an average of 12 SNPs) and annotation of transposons, which are the major contig break regions in these draft genome sequences (Figure 1). Thus, based on phylogenetic and SNP analysis, the outbreak goat (NL3262, 602, CbCVIC1) and human strains (NLhu3345937, 42785537, NL-Limburg) of the CbNL01 genotype cluster together and possess almost identical genomes. Furthermore, these data confirm that goat are the source of the Dutch human Q fever outbreak as suggested previously by genotyping (Figures 1, 2; Roest et al., 2011a).

Both CbNL01 and CbNL12 strains were isolated in The Netherlands during the outbreak period from 2007 to 2010. The CbNL12 genotype strains were the second most prevalent strains after the CbNL01 strains and were only isolated from cattle, goat and sheep, but not from humans. The CbNL12 genome sequence was shown to be similar to the genome sequence of the reference strain NM (Figures 1, 2). Compared to CbNL01 strains, the CbNL12 strains clustered separately based on phylogenomics (cluster 1 and 2, Figure 1) but the distance between these clusters was very small (MUMi distance 0.022–0.03) indicating closely related genomes. Although the CbNL12 and CbNL01 strains belong to distinct MLVA genotypes, the major differences were based on point mutations (average of 2400 SNPs) and the number of transposase encoding genes. The cattle strains 2574 and 701CbB1 used in this study are of the CbNL12 genotype and from different geographical locations. The 701CbB1 strain is an archival sample isolated in France and a similar genotype strain (2574) was recently isolated during the Dutch Q fever outbreak period. The cattle strains were clustered together phylogenetically, indicating a close relationship between these strains. In addition, the published genome sequences of 3 strains (Cb_B1, Cb_B18, EV-Cb_BK10) of the CbNL12 genotype, which were isolated from ruminants in France, were also present in the same cluster (Cluster 2, Figure 1). The presence of similar genome strains obtained from various geographical locations may imply a clonal spread of CbNL12 strains over different European countries. This idea is strengthened by the fact that strains of the CbNL12 genotype were identified in consumer cow milk products obtained from various European countries (Tilburg et al., 2012a). As the CbNL12 strains are not detected in human samples but are spread over many European countries, it could be hypothesized that humans are less susceptible to CbNL12 genotype strains, decreasing its risk for causing an outbreak in humans.

The core genome of sequenced *C. burnetii* strains constitutes up to 75% of the complete gene repertoire of the sequenced *C. burnetii* strains, indicating a conservative nature of evolution. Gene ortholog analysis showed a 98% overlap between strains of the same genotype which is higher than the overlap between strains of the same host-origin. This indicates that strains of the same genotype are of clonal origin with highly conserved gene content (Figure 4). Unique genes were not identified in the Dutch outbreak strains and other closely related strains (CbNL12, Henzerling, and Heizberg), although genotype specific genes were identified (Supplementary Table 2). A search of host-specific genes and signatures (based on point mutation) didn't yield any results, indicating that host-specific genes and mutation were absent. Thus, genotype-specific changes were clearly observed in strains, whereas host-specific changes in the strains could not be observed. Based on these findings, the observed phenotypic differences in clinical manifestations of Q fever in natural hosts, goats (abortion), cattle (rarely clinical symptoms), human acute infections (asymptomatic) and human chronic infections (endocarditis), is most probably due to host-specific (immune) responses to an infection with *C. burnetii* or other complex pathogen-related factors (such as host-tropism) which are not known yet.

The difference between the genome sequences of the Dutch outbreak strains and the reference strain NM were based on point mutations, small deletions in parts of genes and a few deletions of complete genes in the CbNL01 strains (Supplementary Figure 2 and Table 3). Novel genes, potentially associated with increased bacterial virulence, were absent in the CbNL01 strains. This suggests that modifications of existing genes have given rise to the high-virulent features of the outbreak strains rather than the acquisition of novel genetic factors by *C. burnetii*. The present findings support previous observations that genomes of *C. burnetii* lack broad genetic variance, due to absence of DNA exchange machinery and the intracellular lifestyle, which limits opportunities for genetic exchange (Seshadri et al., 2003; Beare et al., 2006; D'Amato et al., 2014). Upon assessing the variants in the Dutch strains, relative to NM, genotype-specific mutations in the CbNL01 strains were predominantly found in hypothetical membrane proteins, transporter proteins, translation proteins, DNA repair protein encoding genes and T4SS genes (Supplementary Figure 2). Our study showed a large number of mutations in membrane protein encoding genes of CbNL01 strains, relative to the CbNL12 strains. Amino acid changes in these membrane proteins may lead to a distinct antigenic profile of the CbNL01 strains, which may result in a change in immune evasion efficiencies. Based on these findings, we speculate that a shift in the bacterial antigenic repertoire has resulted in the increased zoonotic potential of the outbreak strains. Furthermore, mutations in transporter genes involved majorly in metabolite transportation, could be crucial for efficiently assessing host-derived nutrients to adapt in the host hostile environments. Additionally, a large number of mutations in the protein synthesis machinery could be a novel mechanism for adaptation of CbNL01 strains to the host environment. These mutations may result in altered levels of protein synthesis, thereby influencing the growth rate of the bacterium in the host niche (D'Amato et al., 2014). Increased numbers of mutations in DNA-repair genes of the CbNL01 strains could be related to an altered/increased capability of these strains to fight against oxidative stress. This feature might aid in efficient maintenance of the bacterial cell integrity in harsh environments of host cell PV. Finally, the mutations in genes encoding virulence related effector proteins and the T4SS proteins delivering system could contribute to an increased/altered virulence potential of CbNL01 strains by an enhanced/altered protein export. This could be beneficial for *C. burnetii* in effective manipulation of host components or host intracellular processes to enhance successful replication and persistent infections in hosts (Beare et al., 2009).

Our study shows that non-synonymous SNPs in a number of membrane protein encoding genes, transporter genes, genes encoding virulence-related effector proteins and O-antigen genes of CbNL01 strains resulted in gene frame-shifts (Supplementary Figure 2). These frame shifts result in the gain of pseudo-genes, which could be a mechanism of “genome reduction.” It could be speculated that lost gene functions might be compensated by other genes. Alternatively, the bacteria might not require this function anymore due to its intimate relationship with host cells (Merhej et al., 2009). The presence of frame-shift mutations might increase the likelihood of the removal of whole genes by DNA deletion processes (Kuo and Ochman, 2010), as evidenced by the observed deletion of an ankyrin repeat protein ortholog to CBU_0072 in all CbNL01 strains (Supplementary Figure 2). It could be speculated that loss of genes can be a crucial mechanism for specialization of the bacteria to survive in a specific niche as seen previously for other bacterial species (Georgiades and Raoult, 2011; Rolain et al., 2013). On the whole, our findings favor the reductive evolution of genomes in the CbNL01 strains, where increased gene loss could have led to bacterial specialization in a host niche (Georgiades and Raoult, 2011).

In this study we focused on the O-antigen encoding region, the most prominently known virulence-related genomic region of *C. burnetii*, which is prone to deletions resulting in phenotypic phase shifting (Hoover et al., 2002). The sequenced strains were cultivated both in cell-based and in cell-free systems and the passage numbers were kept at the minimum to avoid phenotypic phase shifting. Although we maintained low passage numbers (passages between 4 and 14), the CbNL01 and CbNL12 genotype strains showed partial gene deletions in this region. Such deletions were not observed in the remaining human strains used in this study (Schperling, Scurry, Henzerling, and Heizberg) (Table 3). Deletion of portion of the CBU_0691 and CBU_0686 genes in CbNL01 and CbNL12 strains, respectively were identified. We anticipate that these deletions could have a significant effect on the structure and function of LPS as these genes are primarily involved in LPS biosynthesis. Although, these genes are polymorphic and frameshifted, the Dutch strains (CbNL01: NL3262, 602 and CbNL12: 601) were shown to be virulent in a mice virulence bioassay (Kuley et al., 2015b). Hence, based on these observations the effect of genotype-specific deletions in the LPS encoding region and its effect on virulence of the Dutch strains are not clearly known and should be the further investigated.

Transposable elements are present in *C. burnetii* genomes and contribute to genome plasticity (Beare et al., 2009). The Dutch outbreak strains (NL3262, NL-Limburg) encode more than 100 transposase genes which is the highest number of transposase genes found in *C. burnetii* (30 in NM, Z3055, Dugway; 59 in Cbuk_Q154 and 40 in CbuG_Q212). Although we cannot infer the exact number of transposons in draft genome sequences, we anticipate that the higher number of contigs in the sequenced CbNL01 strains (average of 130) corresponds to the higher number of transposons, as contig ends often map to transposase genes relative to NM genome. The CbNL01 and NM genomes are similar in the majority of their gene content, but differ significantly in genome structure due to a large number of genome rearrangements. Moreover, increased genome arrangements were also seen in NL3262 compared to complete genomes of Z3055, Cbuk_Q154, CbuG_Q212, and Dugway strains (Figure 5). Previous comparative studies of *C. burnetii* complete genomes (Beare et al., 2009) as well as our current analysis showed genome rearrangements at around 0, 21, 6, and 13 chromosomal locations in NM compared to Z3055, Cbuk_Q154, CbuG_Q212, and Dugway strains respectively (Figures 5G–J). Our analysis also showed genome rearrangements of NL3262 at 21, 31, 21, and 23 locations compared to Z3055, Cbuk_Q154, CbuG_Q212, and Dugway strains respectively (Figures 5C–F, Supplementary Table 3). Taken together, comparison of Cbuk_Q154, CbuG_Q212 and Dugway strains showed 40 chromosomal breakpoints relative to NM chromosome and 96 chromosomal breakpoints relative to NL3262 chromosome. This clearly shows high level of rearrangements specific for the outbreak genotype than other *C. burnetii* strains known to date. Around 88% of these chromosomal breakpoints in NL3262 occurred due to homologous recombination of transposons (Figure 5, Supplementary Table 3).

The high number of transposase genes (IS elements) is a unique feature of *C. burnetii* genomes, compared to other obligate intracellular pathogens (*Rickettsia, Chlamydia, Mycobacterium leprae*) and are assumed to be associated with adaptation of the pathogen to different intracellular niches (Seshadri et al., 2003). These IS elements associated with genome rearrangements provide evidence for the notion that the high genome plasticity of the outbreak strains aid in an improved acclimatization of the bacteria in its intracellular niche. However, there is no direct evidence that such large-scale genomic rearrangements have impact on the functionalities of *C. burnetii*. On the other hand, genetic transposition events have been previously suggested to be involved in the generation of genetic variation and adaptation of other bacteria species in various environments (Hall, 1991). Based on the current findings and the literature, it can be hypothesized that the large number of transposons in the outbreak strains might resulted in extensive genome rearrangements in response to environmental challenges, including those in the host. Such rearrangements can result in DNA insertions/deletions, generation of polymorphisms, pseudo-genes (genes disrupted by IS elements) or modulate the gene expression by gene order rearrangements favoring its growth and survival in different niches (Hall, 1991; Seshadri et al., 2003; Beare et al., 2006, 2009; Pallen and Wren, 2007; Rohmer et al., 2007).

In conclusion, the present study provides the first complete study on phylogenetic analysis of Dutch outbreak strains, a few additionally selected strains, and all publicly available *C. burnetii* genome sequences. Hierarchical clustering based on genome content, the *in silico* MLVA genotypes and SNP analysis, showed a genotype-specific clustering of strains, showing highly similar genomes of *C. burnetii* within each genotype. The differences between the Dutch outbreak strains relative to the reference NM and CbNL12 strains were mainly based on single nucleotide polymorphisms. We hypothesize that high numbers of transposons, genotype specific polymorphisms in membrane protein encoding genes and virulence related genes could have resulted in a highly flexible genome, an altered antigenic profile and specialized virulence mechanisms. The observed subtle genetic differences form the basis for the increased epidemic potential and the Q fever outbreak in The Netherlands. Finally, the comparative analysis of several *C. burnetii* strains from this study has the potential to provide information on cross-protective vaccine candidates. Additionally, the consistent genotype-specific genetic differences; such as SNPs and deletions could be used as additional markers for better characterization of *C. burnetii* isolate.

## Author contributions

RK carried out design of study, sample collection, genome sequencing, analysis, interpretation of data and drafting the manuscript. EK was involved in data analysis. HS and HR were involved in interpretation of data and critically revising the manuscript. MS was involved in revising the manuscript. AB carried out conception and design of study, data interpretation and helped to draft the manuscript. All authors read and approved the final manuscript.

### Conflict of interest statement

The authors declare that the research was conducted in the absence of any commercial or financial relationships that could be construed as a potential conflict of interest.
