# Supplementary material for: Genome Plasticity and Polymorphisms in Critical Genes Correlate with Increased Virulence of Dutch Outbreak-Related Coxiella burnetii Strains
Source: Front Microbiol. 2017 Aug 10;8:1526. doi: 10.3389/fmicb.2017.01526 (PMC5554327; doi:10.3389/fmicb.2017.01526)
Supplement: Supplementary file 4 [file DataSheet1.DOCX]

**Supplementary material:**

**Supplementary figure 1:** Edwards-Venn diagrams showing the number of orthologs and unique predicted coding proteins among strains originated from same host species.

Number of ortholog genes shared between A) cattle-derived strains B) goat-derived strains C) human (acute infected patients)-derived strains and D) human (chronic infected patients)-derived strains is represented by Edwards-Venn diagrams. Transposase coding proteins were included in the analysis and pseudogenes were scored as absent.

**Supplementary figure 2:** Heat map showing Genes affected (deleted and mutated) in CbNL01 and CbNL12 strains with respect to the reference strain NM.

**Supplementary table 1**: List of orthologs in sequenced *C. burnetii* along with proteins that are ortholog with reference NM as assessed by proteinortho software.

The non-orthologs with reference NM are represented as “-”. These genes include point mutations and small insertion/ deletion of <50 bp in existing pseudo genes of NM (pseudo genes are not included in the otholog analysis). Annotation of pseudo genes of NM and COG categories of orthologs are mentioned in the table.

**Supplementary table 2:** List of genotype-specific orthologs in sequenced *C. burnetii* strains as assessed by proteinortho software.

Non-orthologs between strains of different genotype as well as non orthologs with reference NM strain are represented as “-”. These genotype-specific genes include point mutations and small insertion/ deletion of <50 bp in existing pseudo genes of NM (pseudo genes are not included in the otholog analysis) or contain partial ORF relative to complete ORF in NM. Pseudo genes and complete ORF of NM are mentioned in the table.

**Supplementary table 3:** *C. burnetii* strains genome syntenic blocks relative to NL3262 and flanking transposases in NL3262 strain.
