# Supplementary figures and images for: Genome Plasticity and Polymorphisms in Critical Genes Correlate with Increased Virulence of Dutch Outbreak-Related Coxiella burnetii Strains
Source: Front Microbiol. 2017 Aug 10;8:1526. doi: 10.3389/fmicb.2017.01526 (PMC5554327; doi:10.3389/fmicb.2017.01526)

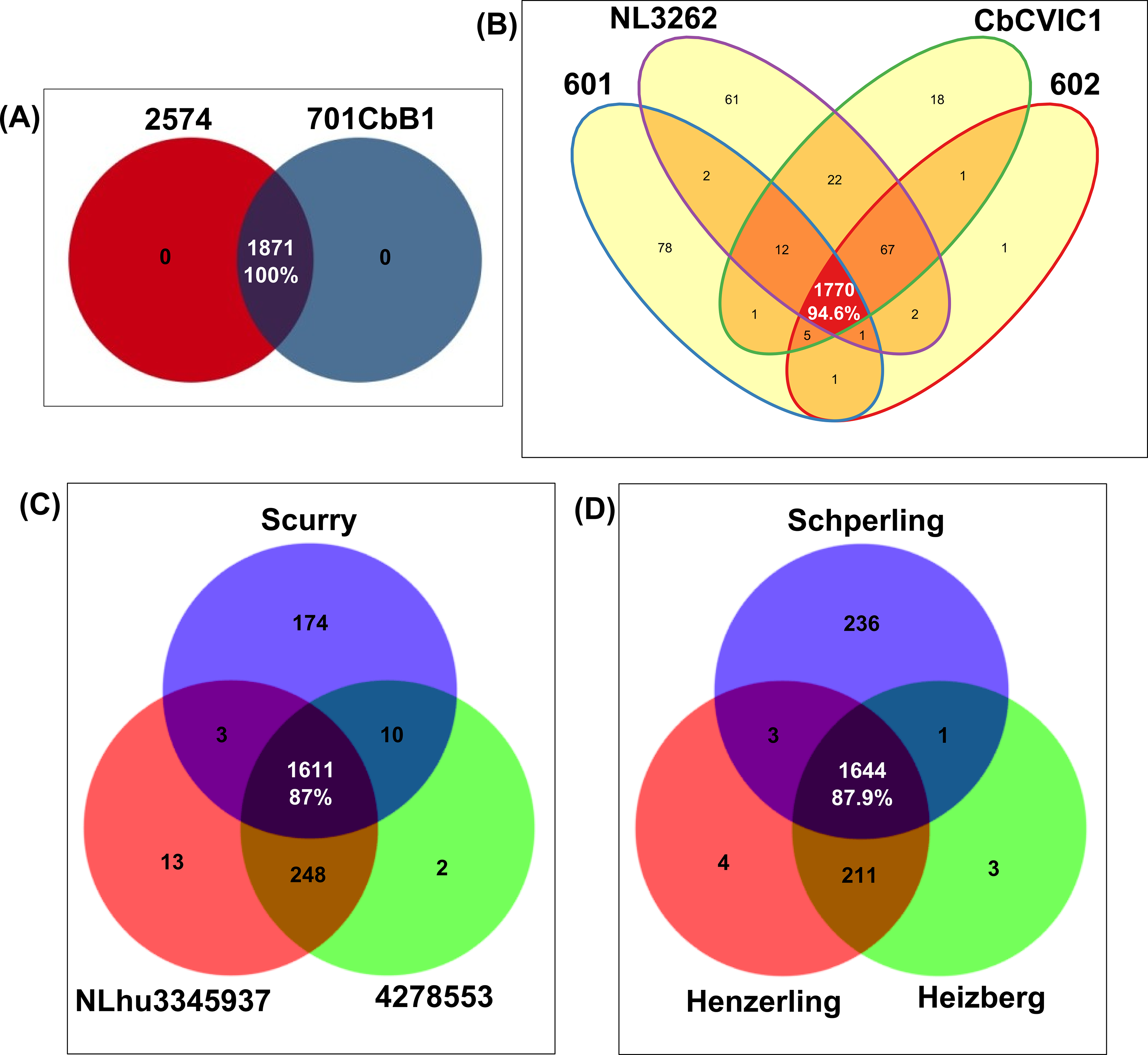

Supplement: Supplementary file 5 [file Image1.TIF]
