# Supplementary material for: Genome Plasticity and Polymorphisms in Critical Genes Correlate with Increased Virulence of Dutch Outbreak-Related Coxiella burnetii Strains
Source: Front Microbiol. 2017 Aug 10;8:1526. doi: 10.3389/fmicb.2017.01526 (PMC5554327; doi:10.3389/fmicb.2017.01526)
Supplement: Supplementary file 6 [file Image2.PDF]

Deleted genes
Mutated genes (Genes with non-synonomous SNP) with respect to Reference NM genome
Non-Mutated genes with respect to Reference NM genome
* frameshifted mutated genes

Category		NL3262	42785537	NLhu3345937	CbCVIC1	602	2574	701CbB1	601	18430	Gene CBU	COG	Gene function
		**CbNL01**					**CbNL12**				**Reference sequence NM**		
Deletion Regions (bp)	9700										1101	-	pseudo genes
											1103	-	Slt family transglycosylase
											1105	-	pseudo genes
											1107	-	pseudo genes
											1111	M	membrane-bound lytic murein transglycosylase A
											1112	L	GIY-YIG catalytic domain protein
	2300										877	-	hypothetical protein
											878	-	pseudo genes
											880	-	hypothetical cytosolic protein
	3600										71	-	pseudo genes
											72	-	ankyrin repeat-containing protein
	2250										16	-	hypothetical protein
											17	-	hypothetical ATPase
											18	-	hypothetical protein
											19	-	hypothetical protein
Membrane proteins											1651	-	hypothetical membrane associated protein
											1819	-	hypothetical membrane associated protein
											577	-	hypothetical membrane associated protein
											837	-	hypothetical membrane associated protein
											850	-	hypothetical membrane associated protein
											866	-	hypothetical membrane associated protein
											1127	-	hypothetical membrane associated protein
											1194	-	hypothetical membrane associated protein
											1202	-	hypothetical membrane associated protein
											1209	-	hypothetical membrane associated protein
											1217	DZ	hypothetical membrane associated protein
											1429	S	hypothetical membrane associated protein
											1559	S	hypothetical membrane associated protein
											1576	-	hypothetical membrane spanning protein
											1800	S	hypothetical membrane spanning protein
											1831	S	hypothetical membrane spanning protein
											1845	-	hypothetical membrane spanning protein
											1920	U	hypothetical membrane spanning protein
											214	R	hypothetical membrane spanning protein
											1910	O	hypothetical membrane spanning protein
											995	-	hypothetical membrane spanning protein
											56	MU	hypothetical membrane spanning protein
											1811	MU	hypothetical membrane spanning protein
											193	-	hypothetical exported membrane spanning protein
											945	-	hypothetical exported membrane spanning protein
											1231	-	hypothetical exported membrane spanning protein
											122	-	hypothetical membrane spanning protein
		*	*	*	*	*					154	NU	hypothetical membrane spanning protein
		*	*	*	*	*	*	*	*	*	184	S	hypothetical membrane spanning protein
											197	S	hypothetical membrane spanning protein
											198	M	hypothetical membrane spanning protein
											398	-	hypothetical membrane spanning protein
		*	*	*	*	*					410	-	hypothetical membrane spanning protein
		*	*	*	*	*					485	-	hypothetical membrane spanning protein
											534	-	hypothetical membrane spanning protein
		*	*	*	*	*					543	-	hypothetical membrane spanning protein
											611	M	hypothetical membrane spanning protein
											617	S	hypothetical membrane spanning protein
		*	*	*	*	*					687	-	hypothetical membrane spanning protein

										914	-	hypothetical membrane spanning protein
										970	-	hypothetical membrane spanning protein
										1042	S	hypothetical membrane spanning protein
										1058	S	hypothetical membrane spanning protein
	*	*	*	*	*					1061a	-	hypothetical membrane spanning protein
	*	*	*	*	*	*	*	*	*	1175	-	hypothetical membrane spanning protein
										1260	-	hypothetical membrane spanning protein
										1263	-	hypothetical membrane spanning protein
										1302	-	hypothetical membrane spanning protein
	*	*	*		*					1369	-	hypothetical membrane spanning protein
	*	*	*	*	*					1370	-	hypothetical outer membrane protein
										1372	-	inner membrane protein oxaA
										1376	M	integral membrane protein
										1395	-	membrane endopeptidase, M50 family
										1413	-	OmpA-like transmembrane domain protein
	*	*	*	*	*	*	*	*	*	1530	-	outer membrane lipoprotein
										1561	-	outer membrane lipoprotein LolB
										1701	-	outer membrane protein
	*	*	*	*	*					1733	-	outer membrane protein
										1814	-	outer membrane protein assembly complex
						*	*	*	*	1818	-	outer membrane protein, OMP85 family protein
										1829	M	putative cytochrome c oxidase, membrane subunit
										1851	-	TolC family type I secretion outer membrane protein
										1863	-	type 4 pili biogenesis protein
										1950	S	type I secretion outer membrane protein
										910	R	hypothetical membrane associated protein
										1333	R	hypothetical membrane spanning protein
										1334	S	hypothetical membrane spanning protein
										1556	-	hypothetical membrane spanning protein
										1884	S	hypothetical exported membrane spanning protein
										278	U	MarC membrane protein
										936	-	OmpA-like transmembrane domain protein
										307	-	OmpA-like transmembrane domain protein
										311	-	outer membrane porin P1
										1190	M	outer-membrane lipoproteins carrier protein
Transporters										728	Q	ABC transporter ATP-binding protein
										776	V	ABC transporter ATP-binding protein
										1952	R	ABC transporter ATP-binding protein
										729	Q	ABC transporter
										933	GM	ABC transporter permease protein
										482	ET	arginine ABC transporter
										1967	G	Bcr/CflA subfamily drug resistance transporter
										107	P	D-methionine ABC transporter, ATP-binding protein
										109	P	D-methionine ABC transporter
										1208	G	major facilitator transporter
	*	*	*	*	*					1859	EP	oligopeptide ABC transporter, permease protein
										364	P	phosphate transporter
										2058	G	proline/betaine transporter
										1066	P	transporter, divalent anion:sodium symporter
										272	G	transporter, MFS superfamily
										432	G	transporter, MFS superfamily
										566	G	transporter, MFS superfamily
										906	G	transporter, MFS superfamily
										1484	G	transporter, MFS superfamily
										1858	E	peptide ABC transporter, permease protein
										2055	P	zinc uptake transporter
	*	*	*	*	*	*	*	*	*	371	G	multidrug resistance transporter, Bcr family
	*	*	*	*	*	*	*	*	*	1244	P	EmrB/QacA family drug resistance transporter
						*	*	*	*	14	P	low-affinity inorganic phosphate transporter
										459	P	transporter, monovalent cation:proton antiporter-2
										504	E	di-/tripeptide transporter
										753	V	AcrB/AcrD/AcrF family transporter
										754	M	RND family efflux transporter MFP subunit
										777	R	ABC transporter permease
										797	G	EmrB/QacA family drug resistance transporter
										803	M	RND family efflux transporter MFP subunit
	*	*	*	*	*					804	V	AcrB/AcrD/AcrF family transporter
										833	V	ABC transporter permease/ATP-binding protein

											856	V	lipid ABC transporter permease/ATP-binding protein
											922	G	transporter, MFS superfamily
											1075	M	transporter
											1130	S	OPT family oligopeptide transporter
											1362	P	cation diffusion facilitator family transporter
											1808	V	export ABC transporter permease protein
											1810	M	ABC transporter
											1896	G	transporter, major facilitator family
											2068	G	transporter, MFS superfamily
											354	E	amino acid permease
											484	E	amino acid ABC transporter, permease protein
											953	E	amino acid permease
											1347	E	amino acid antiporter
											1539	E	tryptophan/tyrosine permease family protein
											1796	E	amino acid permease family protein
											1798	E	amino acid permease family protein
											1093	V	AcrB/AcrD/AcrF family transporter
											481	E	arginine ABC transporter ATP-binding protein
											1179	G	Bcr/CflA subfamily drug resistance transporter
											265	G	glucose/galactose transporter
											649	R	riboflavin transporter
											484	E	amino acid ABC transporter, permease protein
Virulence-related genes	DNA Repair Genes										305	LK	RecG-like helicase
											1083	L	DNA mismatch repair enzyme (predicted ATPase)
											1229	S	hypothetical protein
											1243	L	Exonuclease VII, large subunit
											1422	O	Predicted ATP-dependent serine protease
											1148	LK	Transcription-repair coupling factor
											557	L	DNA polymerase III, delta subunit
											271	L	Single-stranded DNA-binding protein
											274	L	Excinuclease ATPase subunit
											506	L	Single-stranded DNA-specific exonuclease
											542	L	NAD-dependent DNA ligase
											1056	L	Mismatch repair ATPase (MutS family)
											1297	L	ATPase involved in DNA repair
											2054	L	Superfamily I DNA and RNA helicases
											297	L	Exonuclease III
											1795	L	DNA polymerase I
											518	L	Helicase subunit of the DNA excision repair complex
											1185	L	Nuclease subunit of the excinuclease complex
											500	L	ATPase involved in DNA replication
											1337	L	DNA polymerase III, alpha subunit
											1476	K	Transcriptional regulator
											1697	L	Predicted EndoIII-related endonuclease
	Type IV Secretion system										1651	-	hypothetical membrane associated protein
											1650	-	IcmW
											1645	NU	Dot/Icm secretion system ATPase DotB
											1642	-	IcmS
											1631	M	ompA family protein
											1628	S	IcmK
											1627	S	IcmE
											1648	S	DotA protein
											1643	S	DotD
	Eukaryotic-like protein domain containing proteins	*	*	*	*	*	*	*	*	*	A0016	-	CbhE protein
		*	*	*	*	*	*	*	*	*	A0006	-	hypothetical protein
											1206	-	delta(24(24(1)))-sterol reductase
							*	*	*	*	599	P	3'(2'),5'-bisphosphate nucleotidase
											701	P	3'(2'),5'-bisphosphate nucleotidase
											1292	R	ankyrin repeat-containing protein
		*	*	*	*	*	*	*	*	*	1253b	-	ankyrin repeat-containing protein
											175	T	serine/threonine kinase protein
											1136	R	enhanced entry protein enhC
											1457	R	tetratricopeptide repeat family protein
											295	-	hypothetical protein
											1217	DZ	hypothetical membrane spanning protein
											A0013	-	hypothetical protein
							*	*	*	*	1158	-	sterol delta-7-reductase
											547	R	TPR domain-containing protein

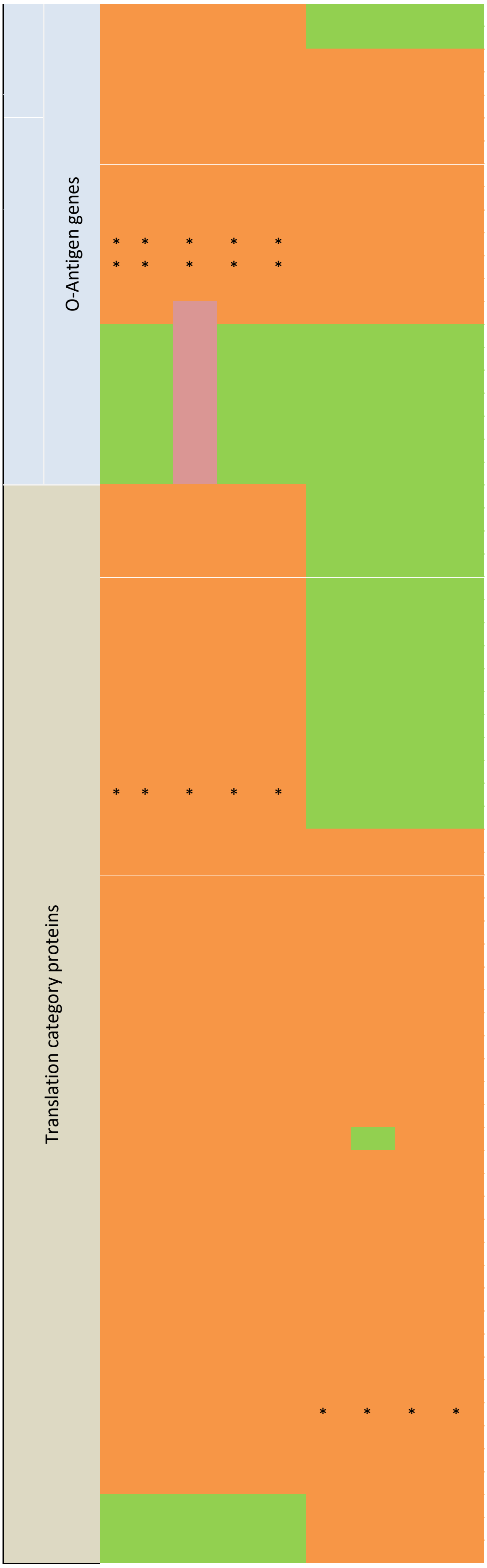

698	-	hypothetical protein
688	MG	GDP-L-fucose synthase
701	P	3'(2'),5'-bisphosphate nucleotidase
700	P	bifunctional sulfate adenylyltransferase subunit 1
695	-	hypothetical protein
694	-	glycosyltransferase
693	C	putative pyruvate dehydrogenase
689	M	GDP-mannose 4,6 dehydratase
685	-	hypothetical protein
706	-	hypothetical protein
687	-	hypothetical exported membrane spanning protein
691	-	methyltransferase
701	P	3'(2'),5'-bisphosphate nucleotidase
678	M	ADP-heptose synthase, putative
676	MG	NAD dependent epimerase/dehydratase
683	-	Pseudo gene
682	-	hypothetical protein
681	MG	hypothetical protein
680	M	UDP-glucose/GDP-mannose dehydrogenase
679	R	oxidoreductase, Gfo/Idh/MocA family
677	M	NAD dependent epimerase/dehydratase family
241	J	50S ribosomal protein L2
253	J	50S ribosomal protein L6
286	J	poly(A) polymerase
487	J	ribosomal large subunit pseudouridine synthase C
757	J	ribosomal large subunit pseudouridine synthase D
808	J	valyl-tRNA synthetase
811	J	peptide chain release factor 3
1052	J	alanyl-tRNA synthetase
1201	J	S-adenosylmethionine
1321	J	phenylalanyl-tRNA synthetase subunit beta
1353	J	23S rRNA Um2552 2'-O-methyltransferase
1430	J	tRNA pseudouridine synthase B
1841	J	peptidyl-tRNA hydrolase
1907	J	acetyltransferase
1982	J	dimethyladenosine transferase
1432	J	translation initiation factor IF-2
234	J	30S ribosomal protein S7
240	J	LSU ribosomal protein L23P
244	J	SSU ribosomal protein S3P
245	J	50S ribosomal protein L16
247	J	SSU ribosomal protein S17P
250	J	50S ribosomal protein L5
257	J	LSU ribosomal protein L15P
260	J	30S ribosomal protein S13
304	J	putative endoribonuclease L-PSP
396	J	isoleucyl-tRNA synthetase
443	J	tRNA (Guanine-N(1)-)-methyltransferase
444	J	16S rRNA-processing protein RimM
486	J	ribonuclease
904	J	tRNA-dihydrouridine synthase A
986	J	RNA methyltransferase
1059	J	RNA pseudouridine synthase family protein
1065	J	2'-5' RNA ligase
1082	J	tRNA delta(2)-isopentenylpyrophosphate transferase
1131	J	RNA methyltransferase
1147	J	tRNA-specific 2-thiouridylase MnmA
1432	J	translation initiation factor IF-2
1475	J	aspartyl/glutamyl-tRNA amidotransferase subunit B
1879	J	peptide deformylase
1914	J	glycyl-tRNA synthetase subunit beta
1915	J	ribosomal RNA small subunit methyltransferase B
2003	J	sua5/YciO/YrdC/YwlC family protein
2008	J	arginyl-tRNA synthetase
1875	J	glutathione synthetase
233	J	30S ribosomal protein S12
852	J	polynucleotide phosphorylase/polyadenylase
81	J	prolyl-tRNA synthetase

			1072	J	queuine tRNA-ribosyltransferase
			1326	J	threonyl-tRNA synthetase
			569	J	tRNA-i(6)A37 thiotransferase enzyme MiaB
			2049	J	tryptophanyl-tRNA synthetase
			622	J	zinc-binding domain-containing protein
